# Sphenoidal Sinusitis

**Published:** 1909-11-13

**Authors:** 


### Laryngology and Rhinology.

### SPHENOIDAL SINUSITIS.

Disease of any of the accessory sinuses of the nose is fairly easy to detect and differentiate when the suppuration is so profuse that matter may be seen to exude from the ostium of the affected cell and to reappear quickly after it has been wiped away. But the difficulty is far greater in cases of what may be termed "latent" sinus disease, in which the quantity of pus secreted is so scanty that it is only to be detected after repeated examinations. These remarks apply with perhaps especial force to cases of suppuration within the sphenoidal sinus and posterior ethmoidal cells, for certain additional signs which aid us in the localisation of disease of the antrum and frontal sinus are not applicable. Thus transillumination cannot be employed, nor can the sinus be explored with trocar and cannula so easily as can the antrum, for removal of part of the middle turbinal is usually necessary before the sphenoid can be brought into view, and this is naturally not to be done unless the presumptive evidence of disease is fairly strong; so also the local tenderness which is so valuable a sign of frontal sinusitis cannot be elicited in disease of the posterior cells. It therefore follows that the diagnosis of sphenoidal sinus suppuration, though aided by the situation of any pain or headache, depends chiefly on the detection and localisation of the discharge, and will in general be easy or difficult according to the profuseness or scantiness of the suppuration.

Suppurative disease of the sphenoidal sinuses is probably a more frequent affection than is generally realised, and many inveterate cases of so-called "post-nasal catarrh" turn out, after thorough investigation, to be due in reality to sphenoidal sinusitis. As in disease of other sinuses, the principal symptoms are pain and discharge; if the ostium is completely or relatively occluded the pain is severe, but when the ostium is freely patent pain may be slight or absent, and the discharge is then the only complaint. In some cases the pain is intermittent, and gradually increases in intensity until sudden relief is experienced, together with a flow of pus; this is very characteristic of any sinus disease, and is due to temporary closing of the ostium. The discharge from the sphenoidal sinus flows downwards into the naso-pharynx, and but little comes from the nostrils; as the ostium is placed high on the anterior wall, it is generally more profuse on stooping, and often also on rising in the morning. The consequences of the discharge of pus into the throat are often apparent, such as pharyngitis and laryngitis, dyspepsia, loss of health and appetite, while affections of the ear are very common as the pus flows over the orifice of the Eustachian tube. The pain usually takes the form of headache, which may be localised to various regions, and is generally bilateral even in affections of one sphenoidal sinus; it is most characteristically felt in the centre of the head, the occipital region, or behind the eyes. Severe neuralgia of the fifth nerve may result from irritation of Meckel's ganglion. Intracranial complications also occur, probably only when the ostium is occluded. Lesions of the optic nerve occur with sufficient frequency to make it advisable to exclude sphenoidal disease in all such cases if no other cause is apparent; such lesions may also be due to disease of the posterior ethmoidal cells, or even of the opposite sphenoidal sinus.

Though the localisation of the pain is of importance, the only method of diagnosis is to see the discharge exuding from the ostium or to obtain pus by probing or tapping the cavity. The discharge from the sphenoidal sinus is seen far back in the nose above and behind the middle turbinal body or trickling through the "olfactory slit" between this body and the septum. Pus which reappears here after wiping away, or which is persistently seen here on several examinations, must come from the sphenoidal or the posterior ethmoidal cells. The diagnosis is made more certainly by posterior rhinoscopy; pus in the naso-pharynx or lying on the upper surface of the palate is of no value for localisation, but if the pus is seen within the choanæ lying above the middle turbinal body it must have been secreted by the posterior cells. It is now usually necessary to remove the posterior half or third of the middle turbinal body in order to get a view of the anterior surface of the sphenoid. It is advisable to do this under general anæsthesia, and, if the bleeding can be controlled by a temporary packing with adrenalin, the sinus may be explored immediately; if this cannot be done, the investigation should be continued on a subsequent occasion, and for this cocaine is usually sufficient. When a good view of the sphenoid has been obtained, a streak of pus may often be seen running down from the ostium; in any case, an attempt is made to enter the ostium with a fine probe which is bent very slightly downwards for the last inch. The ostium is situated near the outer side of the anterior wall and very high up, some 2½ to 3½ inches from the anterior nares and in a line from the latter which crosses the middle of the lower border of the middle turbinal body and forms an angle of about 45° with the floor of the nose. If, as often happens, the ostium cannot be found, the thin anterior wall must be penetrated by a stiff probe or a trocar and cannula. The utmost gentleness and care must be used, the instrument must be kept as nearly horizontal as possible to avoid its slipping upwards, and no curetting or other manipulation within the sinus should be undertaken. If pus is found, the natural or artificial opening is enlarged to admit a special punch, and this is best done with a fine long spoon, then with the punch a large opening is made. For fear of injuring the roof of the sinus, all cutting should be done in a downward direction. The after-treatment consists in cleaning the sinus with peroxide of hydrogen and the use of mild lotions. Recovery is rapid, but, as the opening tends to contract very quickly, it may need to be re-enlarged, or the healing edge may be destroyed with the galvano-cautery.

